# Development and verification of a nomogram for predicting the prognosis of resectable gastric cancer with outlet obstruction

**DOI:** 10.1186/s12885-022-10260-9

**Published:** 2022-11-09

**Authors:** Chengzhi Wei, Changhua Li, Xiaojiang Chen, Guoming Chen, Runcong Nie, Chongyu Zhao, Zhiwei Zhou, Yongming Chen

**Affiliations:** 1 Department of Gastric Surgery, State Key Laboratory of Oncology in South China, Collaborative Innovation Center for Cancer Medicine, Sun Yat-Sen University Cancer Center, Guangzhou, Guangdong China; 2 Department of Gastrointestinal Surgery/Department of Emergency, The First Affiliated Hospital of Guangxi Medical University, Nanning, Guangxi China; 3 Department of Pancreatobiliary Surgery, State Key Laboratory of Oncology in South China, Collaborative Innovation Center for Cancer Medicine, Sun Yat-Sen University Cancer Center, Guangzhou, Guangdong China

**Keywords:** Gastric cancer, Outlet obstruction, Gastrectomy, Nomogram, Overall survival

## Abstract

**Background:**

The prognosis of patients with gastric cancer (GC) with gastric outlet obstruction (GOO) after gastrectomy is highly variable. In this study, we aimed to develop a nomogram to predict the prognosis of these patients.

**Patients and Methods:**

Data from 218 GC patients with GOO who underwent gastrectomy at Sun Yat-sen University Cancer Center were retrospectively collected as a training cohort. The data of 59 patients with the same diagnosis who underwent gastrectomy at the First Affiliated Hospital of Guangxi Medical University were collected as an external verification cohort. A nomogram for the overall survival (OS) was developed using the Cox regression model in the training cohort, which was validated in a verification cohort.

**Results:**

Multivariate analysis showed that the surgical procedure (*P* < 0.001), period of chemotherapy (*P* < 0.001), T stage (*P* = 0.006), N stage (*P* = 0.040), systemic immune-inflammatory index (SII) (*P* < 0.001), and fibrinogen level (*P* = 0.026) were independent factors affecting OS. The nomogram constructed on the aforementioned factors for predicting the 1- and 3-year OS achieved a Harrell’s concordance index (C-index) of 0.756 and 0.763 for the training and verification cohorts, respectively. Compared with the 8^th^ American Joint Committee on Cancer (AJCC) Tumour-Node-Metastasis (TNM) staging system, the nomogram had higher C-index values and areas under the curve (AUCs) and slightly higher net clinical benefit.

**Conclusion:**

Compared to the 8^th^ AJCC staging system, the newly developed nomogram showed superior performance in predicting the survival of GC patients with GOO after gastrectomy.

## Introduction

Gastric cancer (GC) ranks fifth in global cancer incidence and fourth in mortality, with an estimated 769,000 deaths reported in 2020 [1]. Gastric outlet obstruction (GOO) is a severe and complicated complication of advanced GC. GOO causes anorexia, nausea, vomiting, intolerance to oral nutrition, loss of body mass, and subsequent malnutrition, which reduces the quality of life (QOL) and affects the tolerability of cancer treatments [2].

Compared with GC patients without GOO, those with GOO commonly exhibit more aggressive pathologic features and worse nutritional status, as manifested by deeper primary tumor invasion, more lymph node metastasis, more weight loss, and lower prealbumin levels [3]. Several studies have shown GOO to be an independent factor influencing poor prognosis [3–5]. Even after radical gastrectomy, the long-term prognosis of GC with GOO is generally poor, with a reported 5-year survival probability of only 20.4–42.8% [3, 6, 7]. However, in clinical practice, some patients tended to exceed survival expectations due to favorable clinical conditions and slow cancer progression. Consequently, the prognosis of patients with GC with GOO varies greatly. To date, only a handful of studies have assessed prognostic factors for such patients with different biological behaviors and late stages. A recent study identified preoperative performance status, postoperative chemotherapy, improvement in oral intake, and improvement in postoperative QOL as independent prognostic factors in patients with malignant gastric outlet obstruction who underwent palliative surgery [8]. The multivariate survival analysis of 48 patients with stage IV gastric cancer outlet obstruction by Choi et al. showed that palliative chemotherapy was a protective factor, while specific types of hematogenous and lymphatic metastasis were risk factors [9]. To our knowledge, no nomogram model for predicting the prognosis of such patients has been reported, and there remains a lack of suitable and efficient methods for predicting survival.

Therefore, in the present study, we sought to establish a convenient nomogram model with excellent discrimination performance by integrating the clinicopathological factors, treatment modalities, and inflammatory factors for predicting the prognosis of GC with GOO undergoing gastrectomy, to achieve individualized prediction and provide a decision-making basis for highly tailored clinical management.

## Patients and methods

### Patients

A total of 218 GC patients with GOO who underwent gastrectomy at the Sun Yat-sen University Cancer Center from 2001 to 2021 were selected as the training cohort for developing the nomogram. All selected patients satisfied the following inclusion criteria: (i) histologically confirmed primary gastric adenocarcinoma, (ii) complete clinicopathological and follow-up data, (iii) palliative or radical gastrectomy, and (iv) no acute infection or other inflammatory reactions within 2 weeks before blood collection. The exclusion criteria were as follows: (i) inadequate data, (ii) multiple primary GCs, and (iii) other malignant tumors. Fifty-nine patients from the First Affiliated Hospital of Guangxi Medical University who met the aforementioned criteria between 2014 and 2019 were selected as the verification cohort.

### Surgical procedures

Radical gastrectomy with D2 lymphadenectomy was performed in the absence of serious outward invasion of the primary tumor, distant lymph node metastasis, peritoneal metastasis, hepatic metastasis, or other incurable factors. Simultaneously, the margins at both ends of the primary lesion were pathologically negative. Palliative gastrectomy was performed if there was no opportunity for radical gastrectomy.

### Data collection and definitions

All clinicopathological information was obtained from the databases of the two centers, including age, sex, body mass index (BMI), preoperative weight loss, oral intake, performance status (PS), extent of the tumor, TNM stage, tumor differentiation status, tumor size, surgical procedure, intraoperative blood loss, postoperative chemotherapy, and postoperative complications. The following blood parameters were collected: carcinoembryonic antigen (CEA), carbohydrate antigen 19–9 (CA19-9), hemoglobin (HGB), white blood cell count (WBC), platelet count, neutrophil count, lymphocyte count, albumin, fibrinogen, sodium (Na), and potassium (K). All laboratory measurements of peripheral venous blood were performed within 2 weeks before surgery.

The Eastern Cooperative Oncology Group (ECOG) standard was used to calculate the PS. The gastric outlet obstruction scoring system (GOOSS; 0, no oral intake; 1, liquids only; 2, soft solids; and 3, low-residue or full diet) was used to evaluate the oral intake before surgery [10]. The T and N stages were determined based on the 8^th^ American Joint Committee on Cancer (AJCC) Tumour-Node-Metastasis (TNM) classification. The formulas for calculating the systemic immune-inflammatory index (SII) and prognostic nutritional index (PNI) were as follows: SII = platelet × neutrophil/lymphocyte count; PNI = albumin + 5 × lymphocyte count [11].

The continuous variables CEA (5 ng/mL), CA19-9 (27 U/mL), HGB (120 g/L), WBC (9.5 × 10^9^ /L), Na (137 mmol/L), K (3.5 mmol/L), and BMI (18.5 kg/m^2^) were transformed to categorical variables using widely accepted thresholds. The upper limit of the normal value of plasma fibrinogen was different in the two centers at 4 g/L and 5 g/L, respectively; therefore, we divided fibrinogen into normal and elevated groups according to their respective cutoff values. For variables without definite thresholds, such as intraoperative blood loss, SII, and PNI, the optimal cutoff values were determined using version 3.6.1 of X-tile software.

### Follow-up

All follow-up assessments were scheduled by telephone or outpatient visits, every 3 months for the first 2 years, and every 6 months thereafter. The last follow-up date for both the training and verification cohorts was April 2022. We used overall survival (OS) as the endpoint for this nomogram. OS was defined as the time from treatment until death due to any cause or censored at the last follow-up.

### Statistical analyses

The chi-square and Mann–Whitney U tests were performed to determine differences in the binary classification variables and ordinal categorical variables of the two cohorts, respectively. Univariate Cox regression analysis was performed on all variables in the training cohort, and variables with P < 0.05 were included in the multivariate Cox regression model. With the help of the ‘rms’ package in R, statistically significant variables in the multivariate analysis were used to develop the nomogram. Conformity of the nomogram was assessed using a calibration plot method. Predictive performance was measured using the receiver operating characteristic (ROC) curve and Harrell’s concordance index (C-index). Utility was evaluated using a decision curve analysis (DCA). Survival curves were plotted using the Kaplan–Meier method. A two-tailed P-value of < 0.05 was considered statistically significant. All statistical analyses were performed using the X-tile (version 3.6.1), SPSS 26.0, and R (version 4.1.3).

## Results

### Patient characteristics

The clinicopathological characteristics of the patients in the training and verification cohorts are shown in Table 1. The training cohort consisted of 77 (35.3%) females and 141 (64.7%) males with a median age of 59 years. Palliative gastrectomy accounted for 61.0% of the cases (*n* = 133). A total of 152 (69.7%) patients received at least one cycle of postoperative chemotherapy. In the verification cohort, 21 (35.6%) females and 38 (64.4%) males with a median age of 57 years were enrolled. Palliative gastrectomy accounted for 35.6% of the cases (*n* = 21). Forty patients (67.7%) received post-operative chemotherapy. The M stage (*P* < 0.001) and surgical procedure (*P* < 0.001) were statistically different between the two cohorts.

**Table 1:** Clinicopathological characteristics of 277 patients with outlet obstruction in the training and verification cohorts

Variables	Training cohort (%)	Verification cohort (%)	*P*-value
All patients	218	59	
Age (years)			0.494
60	111 (50.9%)	33 (55.9%)	
≥ 60	107 (49.1%)	26 (44.1%)	
Sex			0.969
Male	141 (64.7%)	38 (64.4%)	
Female	77 (35.3%)	21 (35.6%)	
BMI (kg/m^2^)			0.397
18.5	56 (25.7%)	12 (20.3%)	
≥ 18.5	162 (74.3%)	47 (79.7%)	
Preoperative weight loss			0.117
3%	36 (16.5%)	15 (25.4%)	
≥ 3%	182 (83.5%)	44 (74.6%)	
GOOSS			0.341
0/1	103 (47.2%)	32 (54.2%)	
2/3	115 (52.8%)	27 (45.8%)	
PS			0.681
0/1	161 (73.9%)	42 (71.2%)	
2/3	57 (26.1%)	17 (28.8%)	
Extent of tumor			0.474
Distal stomach	184 (84.4%)	52 (88.1%)	
Total stomach	34 (15.6%)	7 (11.9%)	
Surgical procedure			< 0.001
Radical gastrectomy	85 (39.0%)	38 (64.4%)	
Palliative gastrectomy	133 (61.0%)	21 (35.6%)	
Intraoperative blood loss			0.912
< 100 ml	32 (14.7%)	9 (15.3%)	
≥ 100 ml	186 (85.3%)	50 (84.7%)	
Period of chemotherapy			0.758
0	66 (30.3%)	19 (32.2%)	
1–7	101 (46.3%)	27 (45.8%)	
≥ 8	51 (23.4%)	13 (22.0%)	
T stage			0.659
T1-T3	54 (24.8%)	13 (22.0%)	
T4a	115 (52.8%)	32 (54.2%)	
T4b	49 (22.4%)	14 (23.8%)	
N stage			0.175
N0-N2	67 (30.7%)	24 (40.7%)	
N3a	70 (32.1%)	17 (28.8%)	
N3b	81 (37.2%)	18 (30.5%)	
M stage			< 0.001
M0	87 (39.9%)	39 (66.1%)	
M1	131 (60.1%)	20 (33.9%)	
Differentiation status			0.093
Poor	195 (89.4%)	48 (81.4%)	
Well or Moderate	23 (10.6%)	11 (18.6%)	
Tumor size (cm)			0.123
≤ 5	69 (31.7%)	25 (42.4%)	
5	149 (68.3%)	34 (57.6%)	
Complications			0.885
No	190 (87.2%)	51 (86.4%)	
Yes	28 (12.8%)	8 (13.6%)	
CEA (ng/mL)			0.156
5	174 (79.8%)	42 (71.2%)	
≥ 5	44 (20.2%)	17 (28.8%)	
CA19-9 (U/mL)			0.666
27	145 (66.5%)	41 (69.5%)	
≥ 27	73 (33.5%)	18 (30.5%)	
HGB (g/L)			0.897
≤ 120	131 (60.1%)	36 (61.0%)	
120	87 (39.9%)	23 (39.0%)	
WBC (× 10^9^ /L)			0.643
≤ 9.5	200 (91.7%)	53 (89.8%)	
9.5	18 (8.3%)	6 (10.2%)	
SII			0.480
1185.2	165 (75.7%)	42 (71.2%)	
≥ 1185.2	53 (24.3%)	17 (28.8%)	
PNI			0.439
36.5	22 (10.1%)	4 (6.8%)	
≥ 36.5	196 (89.9%)	55 (93.2%)	
Fibrinogen			0.434
Normal	159 (72.9%)	46 (78.0%)	
Elevated	59 (27.1%)	13 (22.0%)	
Na (mmol/L)			0.869
≤ 137	35 (16.1%)	10 (16.9%)	
137	183 (83.9%)	49 (83.1%)	
K (mmol/L)			0.920
≤ 3.5	18 (8.3%)	4 (6.8%)	
3.5	200 (91.7%)	55 (93.2%)	

*BMI* body mass index, *GOOSS* gastric outlet obstruction scoring system, *PS* performance status, *CEA* carcinoembryonic antigen, *CA19-9* carbohydrate antigen 19–9, *HGB* hemoglobin, *WBC* white blood cell count, *SII* systemic immune-inflammatory index, *PNI* prognostic nutritional index, *Na* sodium, *K* potassium

In the training cohort, 139 patients died with a median follow-up of 15.5 months (range, 1.9–145.7 months). The 1- and 3-year OS rates were 67.9% and 29.3%, respectively. In the verification cohort, 32 patients died with a median follow-up of 19.5 months (range, 3.3–70.3 months). The 1- and 3-year OS rates were 78.6% and 31.7%, respectively.

### Independent prognostic factors in the training cohort

The training cohort was used for univariate and multivariate analyses to develop the nomogram further. Univariate analysis identified 12 factors that significantly correlated with OS (Table 2). On this basis, multivariate COX regression analysis defined surgical procedure (*P* < 0.001), period of chemotherapy (*P* < 0.001), T stage (*P* = 0.006), N stage (*P* = 0.040), SII (*P* < 0.001), and fibrinogen level (*P* = 0.026) as independent prognostic factors for OS (Table 2).

**Table 2:** Univariate and multivariate COX regression analysis for overall survival in the training cohort

Variables	Univariate analysis		Multivariate analysis	
	HR (95%CI)	*P*-value	HR (95%CI)	*P*-value
Age (years)		0.527		
60	1			
≥ 60	1.114 (0.797–1.557)			
Sex		0.667		
Male	1			
Female	0.928 (0.652–1.320)			
BMI (kg/m^2^)		0.811		
18.5	1			
≥ 18.5	0.953 (0.644–1.410)			
Preoperative weight loss		0.164		
3%	1			
≥ 3%	0.737 (0.480–1.133)			
GOOSS		0.851		
0/1	1			
2/3	0.968 (0.692–1.355)			
PS		**0.030**		0.148
0/1	1		1	
2/3	1.502 (1.041–2.167)		1.329 (0.904–1.955)	
Extent of tumor		**0.002**		0.067
Distal stomach	1		1	
Total stomach	1.980 (1.283–3.057)		1.586 (0.967–2.601)	
Surgical procedure		** < 0.001**		** < 0.001**
Radical gastrectomy	1		1	
Palliative gastrectomy	2.975 (2.034–4.351)		6.602 (2.385–18.273)	
Intraoperative blood loss		0.062		
< 100 ml	1			
≥ 100 ml	1.667 (0.975–2.852)			
Period of chemotherapy		** < 0.001**		** < 0.001**
0	1		1	
1–7	0.664 (0.455–0.967)	0.033	0.394 (0.257–0.605)	< 0.001
≥ 8	0.357 (0.222–0.573)	< 0.001	0.222 (0.130–0.377)	< 0.001
T stage		** < 0.001**		**0.006**
T1-T3	1		1	
T4a	3.176 (1.895–5.322)	< 0.001	1.865 (1.058–3.287)	0.031
T4b	5.020 (2.837–8.885)	< 0.001	2.854 (1.496–5.445)	0.001
N stage		**0.024**		**0.040**
N0-N2	1			
N3a	1.555 (1.009–2.395)	0.045	1.702 (1.082–2.678)	0.021
N3b	1.779 (1.168–2.710)	0.007	1.707 (1.060–2.748)	0.028
M stage		** < 0.001**		0.150
M0	1		1	
M1	2.633 (1.820–3.809)		0.505 (0.199–1.281)	
Differentiation status		0.508		
Poor	1			
Well or Moderate	0.830 (0.477–1.443)			
Tumor size (cm)		**0.008**		0.497
≤ 5	1		1	
> 5	1.659 (1.144–2.407)		0.868 (0.577–1.306)	
Complications		0.379		
No	1			
Yes	1.243 (0.765–2.018)			
CEA (ng/mL)		**0.025**		0.372
5	1		1	
≥ 5	1.584 (1.060–2.367)		1.216 (0.792–1.868)	
CA19-9 (U/mL)		0.058		
27	1			
≥ 27	1.400 (0.988–1.983)			
HGB (g/L)		0.229		
≤ 120	1			
120	0.808 (0.571–1.144)			
WBC (× 10^9^ /L)		0.100		
≤ 9.5	1			
9.5	1.618 (0.912–2.868)			
SII		**0.004**		** < 0.001**
1185.2	1		1	
≥ 1185.2	1.720 (1.186–2.495)		2.062 (1.394–3.050)	
PNI		**0.018**		0.518
36.5	1		1	
≥ 36.5	0.547 (0.332–0.901)		0.839 (0.493–1.428)	
Fibrinogen		**0.008**		**0.026**
Normal	1		1	
Elevated	1.637 (1.139–2.351)		1.553 (1.054–2.287)	
Na (mmol/L)		0.301		
≤ 137	1			
137	0.793 (0.510–1.232)			
K (mmol/L)		0.195		
≤ 3.5	1			
3.5	0.685 (0.386–1.214)			

*HR* hazard ratio, *CI* confidence interval, *BMI* body mass index, *GOOSS* gastric outlet obstruction scoring system, *PS* performance status, *CEA* carcinoembryonic antigen, *CA19-9* carbohydrate antigen 19–9, *HGB* hemoglobin, *WBC* white blood cell count, *SII* systemic immune-inflammatory index, *PNI* prognostic nutritional index, *Na* sodium, *K* potassium

### Development and verification of the Nomogram

**Fig. 1:**
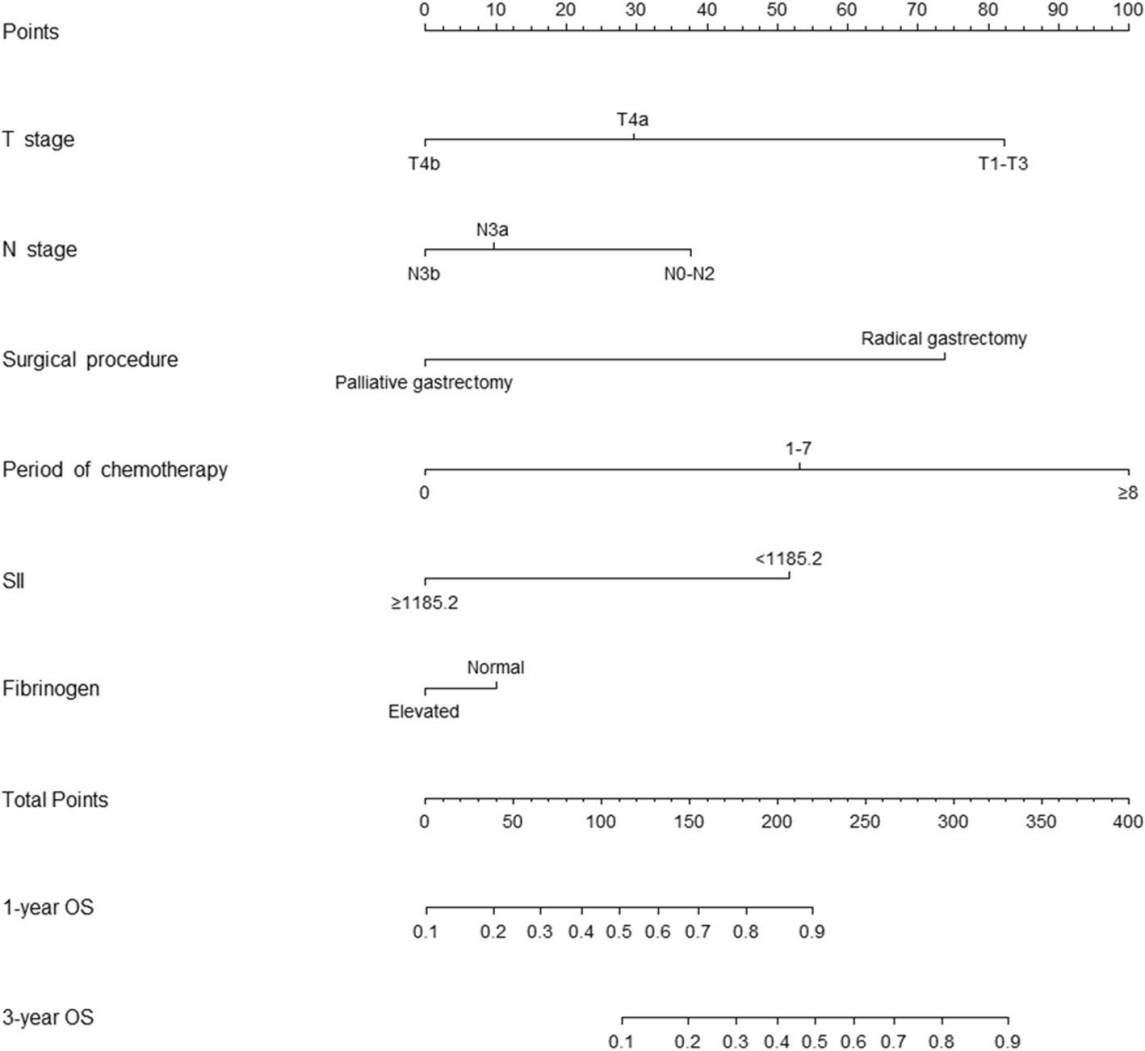
Nomogram for predicting the 1- and 3-year overall survival rates in gastric cancer with outlet obstruction

**Fig. 2:**
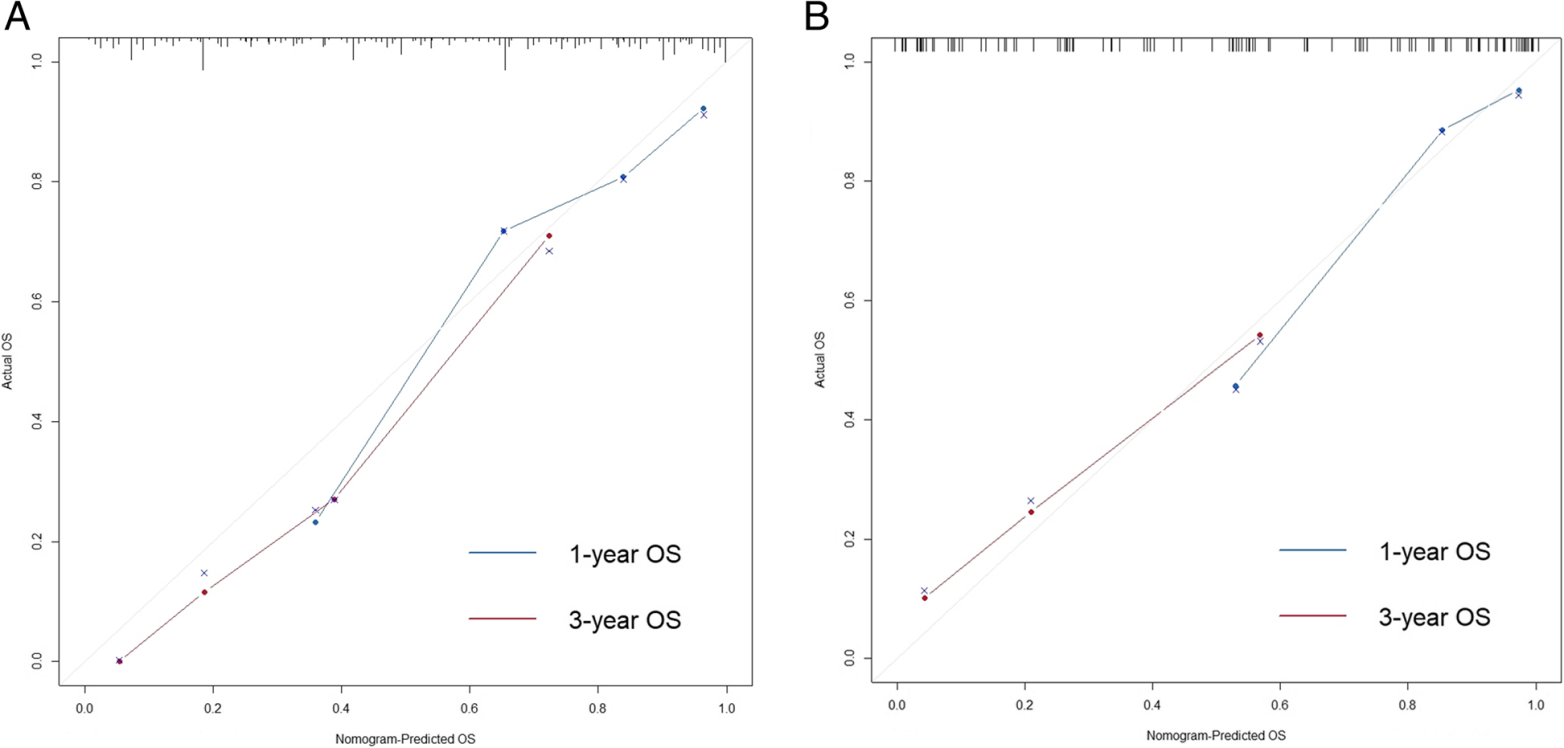
Calibration plots of the nomogram predicting 1- and 3-year overall survival for the training cohort (**A**) and verification cohort (**B**)

Based on the independent factors identified in the multivariate analysis, a nomogram was developed to predict the 1- and 3-year OS rates for GC with GOO (Fig. 1). This nomogram could provide an estimate of the probability of survival outcomes by summing the scores of the six predictors. A higher summed score indicated a better predicted survival outcome. In the calibration plots for both training and verification cohorts (Fig. 2A, B), objectively good consistency between the actual and predicted survival rates could be observed.

### Performances of the Nomogram

**Fig. 3:**
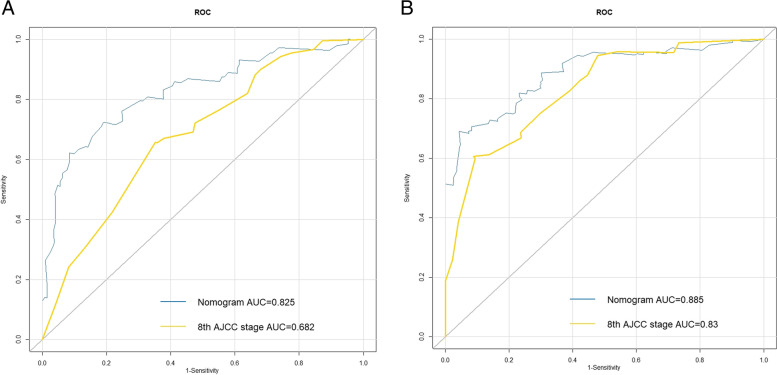
Comparisons of receiver operating characteristic (ROC) curves of the nomogram and the 8^th^ AJCC staging for 1-year (**A**) and 3-year (**B**) overall survival in the training cohort

**Fig. 4:**
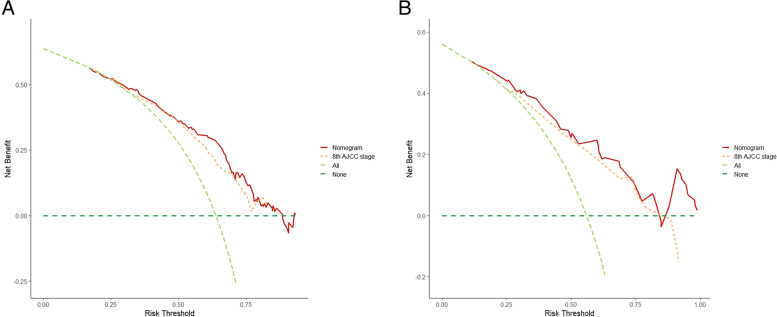
Comparisons of decision curve analysis (DCA) of the nomogram and the 8^th^ AJCC staging for predicting overall survival in the training cohort (**A**) and verification cohort (**B**)

Based on ROC analysis of the training cohort, for the nomogram model and the 8^th^ AJCC staging system, the areas under the curve (AUCs) at 1 year were 0.825 and 0.682, respectively (Fig. 3A), and the AUCs at 3 years were 0.885 and 0.83, respectively (Fig. 3B). The nomogram model demonstrated a C-index of 0.756 (95% CI:0.712–0.800) compared with 0.669 (95% CI:0.619–0.719) for the 8^th^ AJCC staging. In the verification cohort, the C-index of the two were 0.763 (95% CI:0.667–0.859) and 0.675 (95% CI:0.580–0.770), respectively. Moreover, DCA showed that the net clinical benefits of the nomogram slightly outperformed the 8^th^ AJCC staging in the training and verification cohorts within most threshold ranges (Fig. 4A, B). That is, the AUCs, C-index and net clinical benefits of the developed model were higher than those of the 8^th^ AJCC staging.

**Fig. 5:**
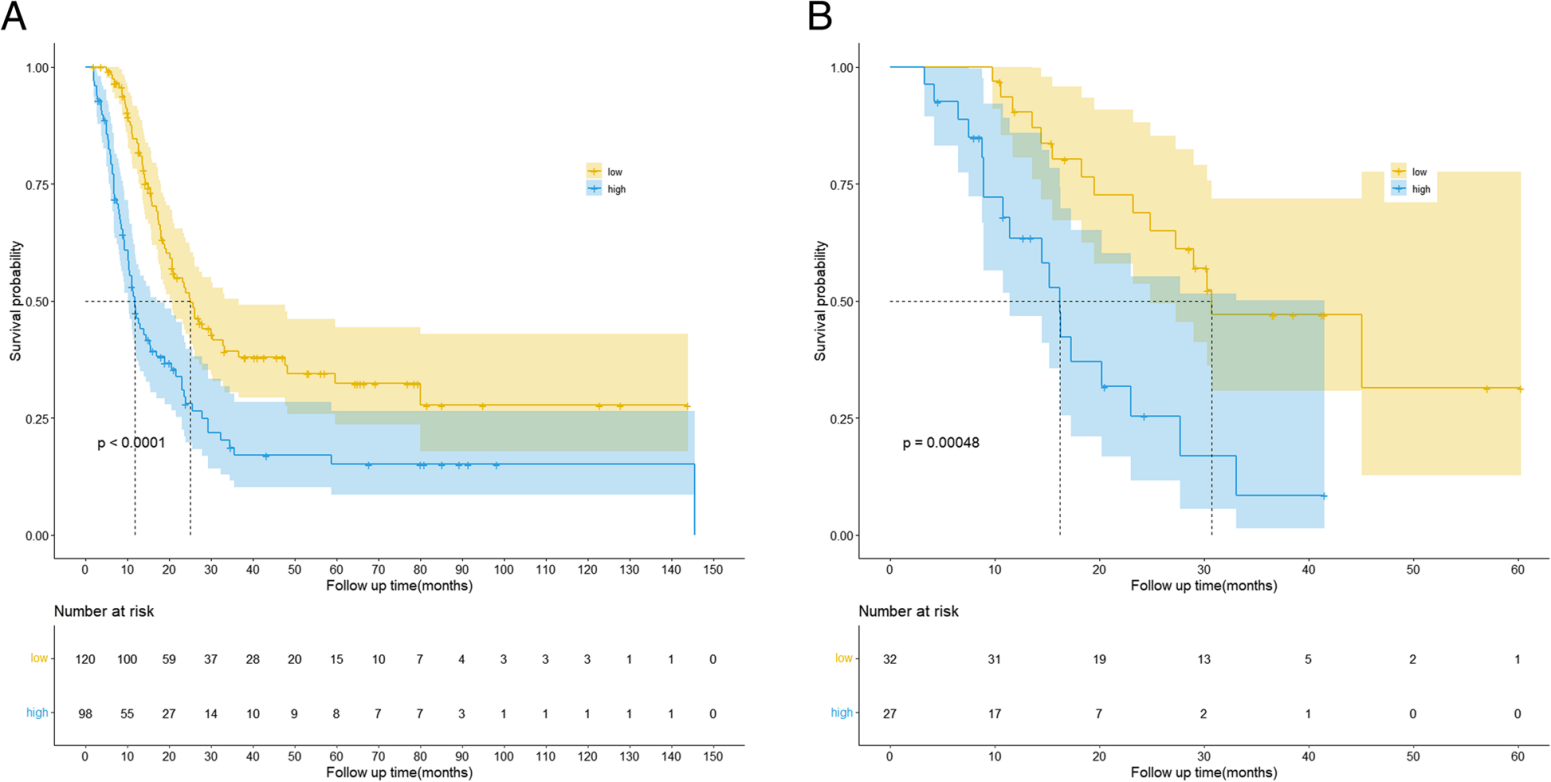
Kaplan–Meier curves for patients stratified by the nomogram score in the training cohort (**A**) and verification cohort (**B**)

The total nomogram score of each patient in the training cohort was calculated, and the cutoff point was selected using the X-tile software. All patients enrolled in our study were stratified into low- and high-risk groups according to a cutoff point of 179. The differences in the OS between the low- and high-risk groups were significant in both the training (*P* < 0.0001) (Fig. 5A) and verification cohorts (*P* = 0.00048) (Fig. 5B), demonstrating the good discrimination ability of our nomogram.

## Discussion

GOO is a common complication of advanced GC. Accurate prediction of the prognosis of GC with GOO is of far-reaching significance for individualized treatment decisions. However, the traditional AJCC staging system cannot provide refined prognostic risk stratification. Considering that treatment and systemic inflammatory response are potential factors influencing survival, it is necessary to develop an accurate and convenient prognostic tool that integrates multiple factors in this population. Through univariate and multivariate Cox regression analyses, our study defined surgical procedure, period of chemotherapy, T stage, N stage, SII, and fibrinogen level as independent factors affecting prognosis. Based on these accessible and objective variables, a nomogram was developed to forecast the 1- and 3-year OS probabilities for resectable GC patients with GOO. Using this nomogram with good performance in terms of calibration, discrimination, and clinical utility, we demonstrated that OS can be more precisely predicted than using the 8^th^ AJCC TNM staging system. Furthermore, the reliability and generalizability of the nomogram were verified using an external cohort.

AJCC staging remains the basic tool for predicting the prognosis of GC. Our nomogram incorporated T and N stages as independent scoring items. Given that GC with GOO has a deeper infiltration depth and a higher status of lymph node metastasis, we categorized the T stage as T1-3, T4a, and T4b, and categorized the N stage as N0-2, N3a, and N3b. Unfortunately, the M stage did not show a significant difference in the multivariate survival analysis. This was related to the addition of the surgical procedure factor. Patients undergoing palliative gastrectomy included not only all M1 patients, but also a minority of M0 patients, which resulted in a higher HR value for the surgical procedure than for the M stage in the multivariate analysis. Zu et al. reported that lymph node metastasis, depth of invasion, curability, and other factors were independent prognostic factors for GC with GOO, similar to our results.[5]

The priority of treatment for GC with GOO is to alleviate obstruction. As a commonly used intervention measure, gastrectomy is classified as either radical resection or palliative resection, according to curability. Radical gastrectomy is indicated for resectable non-metastatic GC. When there are incurable factors, the Japanese treatment guidelines recommend that palliative resection be applied to GC with GOO on the premise of guaranteed surgical safety.[12] Surgical palliation maintained QOL in patients with GOO caused by advanced GC while increasing solid food intake with acceptable safety.[13] The resulting improvements in QOL and intake have been confirmed to translate into survival benefits.[8]

For advanced GC patients undergoing radical gastrectomy, postoperative adjuvant chemotherapy has been recommended as a standard of care according to the Japanese and Chinese guidelines.[12, 14] For GC patients with GOO who have lost the chance of radical gastrectomy, recent studies have found that chemotherapy, rather than the selection of different palliative surgical methods, prolonged survival.[8, 15, 16] A multivariate survival analysis of 104 patients with GOO by Terashima et al.[8] showed that palliative gastrectomy was not superior to gastrojejunostomy in improving OS, and whether receiving postoperative chemotherapy affected the prognosis independently. Chen et al.[15] retrospectively analyzed the efficacy of palliative gastrectomy and gastrojejunostomy using the propensity score matching method and found that there was no statistically significant difference in the median OS between the two groups (8.50 months vs. 11.87 months; *P* = 0.243), while the median OS of patients who received postoperative chemotherapy was significantly longer than those who did not (17.53 months vs. 6.13 months; *P* < 0.001). Similarly, our study demonstrated that postoperative chemotherapy was beneficial to the OS of patients with GOO. As shown in our nomogram, the period of chemotherapy was positively related to OS, with the highest score indicating that it was a powerful predictor. Additionally, this suggests that patients with low nomogram-based scores need to receive as many periods of chemotherapy as possible after surgery for better survival. Therefore, creating optimal conditions and opportunities for chemotherapy, while alleviating GOO, appears to be an important strategy for improving survival.

Increasing evidence has demonstrated that inflammation is a key mediator in tumorigenesis, tumor progression, tumor metastasis, and is an important component of the tumor microenvironment.[17–20] As a biomarker representing the grade of the systemic inflammation response, SII has been proven to be a useful prognostic predictor for various solid tumors.[21–24] Regarding GC, a recent meta-analysis suggested that an elevated pre-treatment SII was associated with later T stage, lymph node metastasis and larger tumor size, and predicted poor OS but not poor disease-free survival (DFS) [25]. Furthermore, SII was a superior predictor of OS in GC and gastroesophageal adenocarcinoma compared with the neutrophil–lymphocyte ratio (NLR) and platelet-lymphocyte ratio (PLR) [26, 27]. Our study confirmed that SII was equally applicable to the prediction of OS in GC with GOO.

Fibrinogen, a coagulation factor closely associated with inflammatory response, was another contributor to this nomogram. Cheng’s meta-analysis of 11 studies demonstrated that an elevated plasma fibrinogen level could predict worse survival in GC and was a risk factor for deeper tumor invasion, lymph node metastasis, and distant metastasis.[28] Previous studies have shown that fibrinogen may promote tumor progression by regulating inflammation, inducing epithelial-mesenchymal transition (EMT), and facilitating angiogenesis.[29–32] This may be a plausible explanation for the potential mechanisms by which plasma fibrinogen levels are associated with prognosis. In our study, there was a difference in the upper limit of the normal value of plasma fibrinogen between the two centers. Considering that the difference was mainly caused by different detection methods, we followed their respective cutoff values to reduce the bias caused by the detection methods. Ultimately, fibrinogen was identified as an independent factor in our prediction model with a score of 10 points. Together with the SII, it provided prognostic information beyond the AJCC staging for GC with GOO.

This study has several limitations. First, the baseline characteristics of the two cohorts were not identical, which may be attributed to the different proportions of hospitalized patients and referral bias between the two centers. Second, patients who underwent gastrojejunostomy and endoscopic stent placement were not included in this study because of incomplete pathological data. Third, the robustness of our nomogram model was restricted by its small sample size.

## Conclusion

A nomogram model predicting the 1- and 3-year OS of patients with GC with GOO after gastrectomy was developed. This nomogram showed superior performance over the 8^th^ AJCC staging system, and it was also a convenient and inexpensive prognostic tool that could inform highly tailored clinical management. Additionally, postoperative chemotherapy is recommended to improve survival of these patients.

## Data Availability

The datasets used and analysed during the current study are available from the corresponding author on reasonable request.
